# Clinical efficacy of sitafloxacin-containing regimens for *Mycobacterium avium* complex pulmonary disease

**DOI:** 10.1186/s12890-025-04004-1

**Published:** 2025-11-18

**Authors:** Naohisa Urabe, Susumu Sakamoto, Nozomi Tokita, Takumi Kanokogi, Rio Tsunehara, Kazuma Kishi

**Affiliations:** Department of Respiratory Medicine, Omori Medical Center, Toho University, 6-11-1 Omori-nishi, Ota-ku, Tokyo, 143-8541 Japan

**Keywords:** *Mycobacterium avium* complex, Sitafloxacin, Fluoroquinolone, Treatment outcome, Refractory MAC-PD

## Abstract

**Background:**

Fluoroquinolones (FQ) have shown efficacy against *Mycobacterium avium* complex (MAC) across experimental settings, in vitro and in vivo. Sitafloxacin (STFX) has demonstrated particularly strong anti-MAC activity, but clinical data on its effectiveness in treating MAC pulmonary disease (MAC-PD) are sparse. This study aimed to evaluate the efficacy of STFX-containing regimens in patients with MAC-PD.

**Methods:**

This retrospective cohort study included 50 patients with MAC-PD who received STFX-containing regimens for ≥6 months at a single center between January 2015 and March 2024. Patients were categorized into four groups: Group 1, STFX-treated without surgery (n = 49); Group 2, STFX started ≥6 months after guideline-based treatment (GBT) began (n = 40); Group 3, poor radiologic response to GBT (n = 38); and Group 4, persistent positive sputum cultures at STFX initiation (n = 19). Primary outcomes assessed at six months included radiologic improvement using the NICE (Nodule, Infiltration, Cavity, Ectasis) score, sputum culture conversion (defined as ≥2 consecutive negative cultures obtained ≥4 weeks apart), and symptom improvement (using the COPD Assessment Test [CAT] score).

**Results:**

Radiologic improvement, symptomatic improvement, and sputum culture conversion were observed in 18.4%, 19.1%, and 20.0% of patients in Group 1; 12.5%, 20.0%, and 12.5% in Group 2; 13.2%, 18.4%, and 13.3% in Group 3; and 5.3%, 15.8%, and 12.5% (2 of 16 evaluable patients) in Group 4, respectively. The two patients who achieved culture conversion had clarithromycin-susceptible strains, non-cavitary disease, and received concomitant ethambutol.

**Conclusions:**

STFX-containing regimens demonstrated modest and overall limited efficacy in patients with MAC-PD, with fewer than 20% achieving radiologic, symptomatic, or microbiological improvement across all groups. STFX may be considered an alternative adjunct when standard therapies are not feasible; however, its overall therapeutic role appears limited.

**Supplementary Information:**

The online version contains supplementary material available at 10.1186/s12890-025-04004-1.

## Background

The incidence of pulmonary disease caused by *nontuberculous mycobacteria* (NTM) has been increasing globally [1]. *Mycobacterium avium* complex (MAC) is the most common causative agent of NTM lung disease in Japan [2]. Guideline-based treatment (GBT) for MAC pulmonary disease (MAC-PD)—consisting of a macrolide (clarithromycin [CLA] or azithromycin [AZM]), ethambutol (EB), and rifampin (RFP)—was first recommended in the 1997 American Thoracic Society (ATS) guidelines [3]. The 2020 ATS/European Respiratory Society/European Society of Clinical Microbiology/Infectious Disease Society of America (ATS/ERS/ESCMID/IDSA) guidelines still endorse this three-drug regimen as standard therapy [4]. Despite long-standing clinical use, GBT yields a treatment success rate of only approximately 60% [5], and patients who fail to achieve sputum culture conversion after six months of GBT are considered to have refractory MAC-PD. For these patients, the addition of aminoglycosides is recommended, of which amikacin liposome inhalation suspension (ALIS) has the strongest supporting clinical evidence. Its treatment success rate, however, is around only 30% [6, 7]. This underscores the urgent need for viable alternative therapeutic options for this difficult-to-treat condition.

Fluoroquinolones (FQ) have demonstrated in vitro and in vivo activity against MAC pathogens [8, 9], and certain agents in this class have shown promise as components of initial treatment regimens [10, 11]. Notably, sitafloxacin (STFX) has been reported to exhibit more potent anti-MAC activity than other FQ [9]. Nevertheless, the clinical utility of STFX in the treatment of MAC-PD remains unclear, and the 2020 guidelines do not include STFX as a recommended treatment option. Therefore, the objective of this study was to evaluate the clinical efficacy of STFX-containing regimens in patients with MAC-PD.

## Methods

### Study population

This single-center retrospective cohort study included 50 patients diagnosed with MAC-PD who received antimicrobial therapy regimens inclusive of STFX for at least six consecutive months at Toho University Omori Medical Center between January 2015 and March 2024. All patients satisfied the ATS/ERS/ESCMID/IDSA guidelines diagnostic criteria for MAC-PD [4]. The cohort included both newly diagnosed and previously treated (recurrent) cases: 39 newly diagnosed and 11 recurrent after prior treatment. Patients who had co-infection with non-MAC NTM or other fungi were excluded.

### Study design

**Fig. 1:**
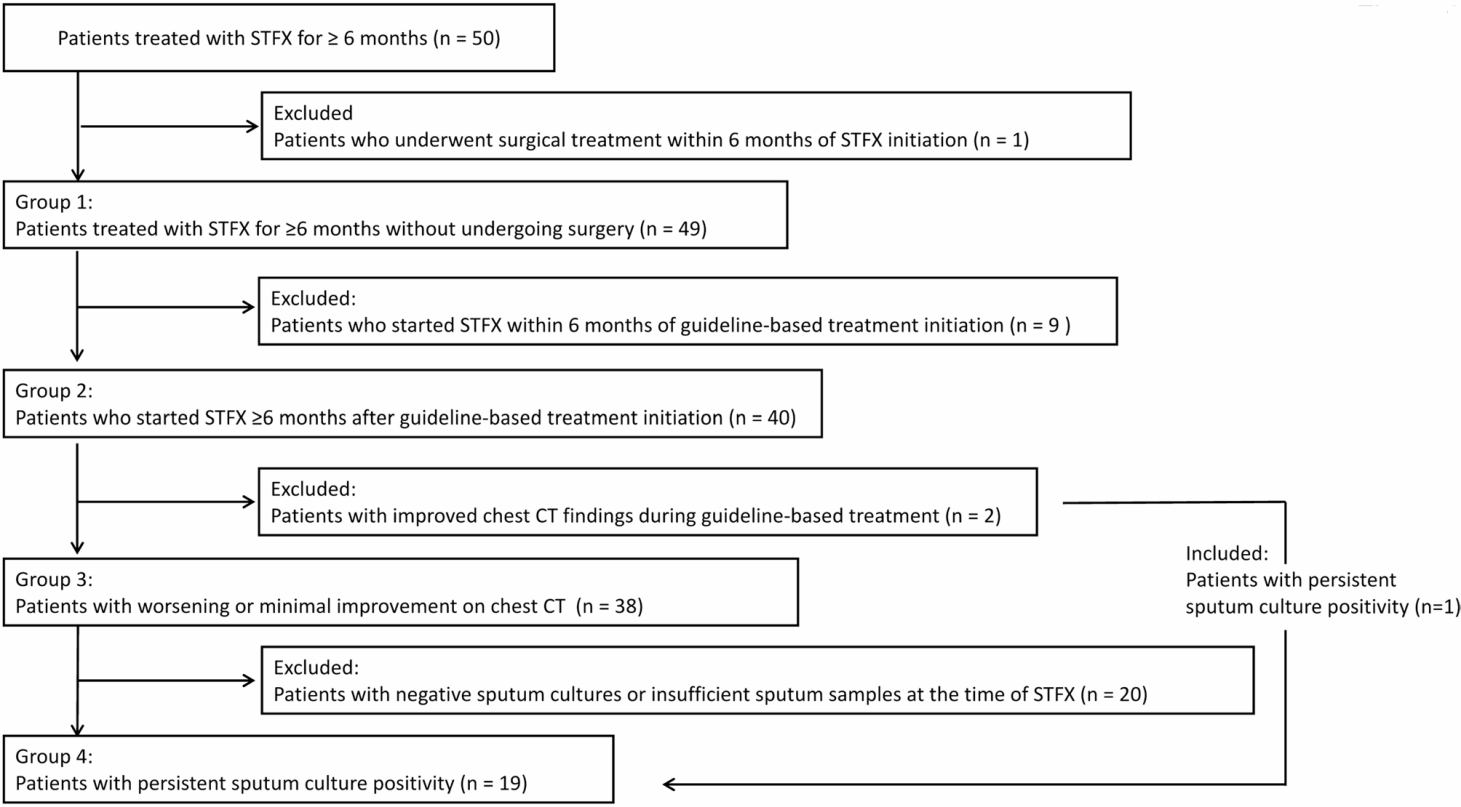
Flowchart of patient selection and classification into four groups based on clinical criteria. STFX: sitafloxacin

We retrospectively reviewed the clinical indications for initiating STFX, the timing of initiation, and the duration of treatment. Based on the timing and indication for STFX initiation, patients were classified into four groups (Fig. 1). Group 1: Patients who received STFX for ≥ 6 months without undergoing surgical resection. Group 2: Patients for whom STFX was initiated ≥ 6 months after the start of GBT. Group 3: Patients with worsening or no improvement in chest CT findings while receiving GBT. Group 4: Patients with persistently positive sputum cultures at the time of STFX initiation. For each group, treatment outcomes were assessed at six months after STFX initiation, including sputum culture conversion, radiologic improvement on chest computed tomography (CT), and improvement in patient-reported symptoms.

In addition, within Group 1, we analyzed the characteristics of patients who demonstrated radiologic improvement on chest CT following an STFX-containing regimen. Factors associated with radiologic improvement were evaluated using univariate logistic regression analyses. We also assessed the characteristics of patients who achieved sputum culture conversion. In Group 4, serial changes in sputum culture results were evaluated to assess microbiological response over time.

### Definitions

Sputum specimen collection was frequently incomplete in this retrospective cohort; therefore, the conventional definition of culture conversion (three consecutive negative cultures) could not be applied in many cases. As shown in Table 8, only 6 of 19 patients had ≥ 3 sputum specimens obtained between 6 and 12 months after STFX initiation. Therefore, culture conversion was defined as ≥ 2 consecutive negative cultures obtained ≥ 4 weeks apart. Radiologic improvement was assessed using the NICE (Nodule, Infiltration, Cavity, Ectasis) scoring system [12], with a decrease in score indicating improvement. Subjective symptom improvement was evaluated using the COPD Assessment Test (CAT) score [13]; a reduction of ≥ 3 points, corresponding to the minimal clinically important difference (MCID) as defined in previous studies on bronchiectasis, was considered clinically meaningful improvement [14]. Based on the Clinical and Laboratory Standards Institute (CLSI) M24A standard, clarithromycin (CLA) resistance was defined as a minimum inhibitory concentration (MIC) ≥ 32 µg/mL [15].

### Data collection

We collected data on patient age, sex, body mass index (BMI), smoking history, comorbidities, concomitant medications, chest CT findings, sputum culture results, drug susceptibility testing of MAC isolates, indications for initiating STFX or reasons for discontinuing, and CAT scores. At our institution, all patients with MAC-PD—including those treated with STFX—routinely undergo chest CT scans and CAT score assessments approximately six months after treatment initiation to assess therapeutic response. The data used in this study were retrospectively collected from these evaluations, which were performed as part of standard clinical care. Baseline chest CT scans were performed within 3 months prior to STFX initiation, and follow-up CT scans used to assess treatment response were obtained between five and seven months after starting STFX. All relevant data are summarized in Supplementary Table 1.

### Statistical analysis

Data are presented as number of patients (percentage), and age is expressed as median with interquartile range. Associations between categorical and continuous variables across the two groups were tested using the Chi-squared or Fisher’s exact test, and the Mann–Whitney U test, respectively. Factors independently associated with radiologic improvement were evaluated using univariate logistic regression analyses. A p-value of < 0.05 was considered indicative of statistical significance. Statistical analyses were performed using SPSS software version 25 (IBM Corp., Armonk, NY).

## Results

### Patient characteristics

A total of 50 patients with MAC-PD were treated with STFX-containing regimens for ≥ 6 months. As summarized in Table 1, the median age was 68 years, and 82% were female. Radiologic patterns included non-cavitary nodular bronchiectasis (NBE) in 56%, cavitary NBE in 32%, and fibro-cavitary (FC) type in 12%. Clarithromycin resistance was identified in 14% (6/43) of isolates tested. Table 2 presents the primary indications for initiating STFX. These include worsening chest CT findings (56%) and lack of radiologic improvement (22%), persistent sputum culture positivity (40%), with some patients meeting multiple criteria. Additional reasons included the need for initial intensive therapy (4%) and discontinuation of EB (12%) or rifampin (RFP) (6%) due to intolerance. STFX was started as part of the initial regimen in 14%, and after ≥ 6 months of GBT in 82% (median, 14.7 months). At the time of data cutoff, 44% of patients remained on treatment. Among those who had discontinued therapy, the median treatment duration was 12.2 months. As shown in Table 3, most patients received either a three-drug (54%) or a two-drug (40%) regimen. All regimens included a macrolide; the commonly used concomitant medications were EB (88%) and RFP (70%).

**Table 1:** Clinical characteristics of patients treated with sitafloxacin

Characteristic		No. of patients (*n* = 50)
Age, years; median (range)^a^		68 (61.3–75)
Sex, female; n (%)		41 (82)
BMI (kg/m^2^); median (range)^a^		18.3 (16.5–19.6)
Smoking, never; n (%)		35 (70)
Comorbidities; n (%)		
Rheumatoid arthritis		5 (10)
Sinusitis		3 (6)
Malignancy		1 (2)
Underlying pulmonary disease; n (%)		
Emphysema		3 (6)
Interstitial pneumonia		3 (6)
Concomitant drug; n (%)		
Corticosteroids		3 (6)
Immunosuppressant		2 (4)
Biopharmaceutical		2 (4)
Infective MAC isolate; n (%)		
*M. avium*		41 (82)
*M. intracellulare*		9 (18)
Subjective symptoms (range)^a^		
CAT score		11 (6–18)
Cough score^b^		2 (1–3)
Sputum score^b^		1 (0–3)
Radiographic pattern; n (%)		
Non-cavitary NBE type		28 (56)
FC type		6 (12)
Cavitary NBE type		16 (32)
Total chest CT score; median (range)^a^		9 (7–12)
Cavitary lesion; n (%)		22 (44)
Proportion of resistant strains; n (%)		
Clarithromycin		6/43 (14)
BMI, Body Mass Index; MAC, *Mycobacterium avium* complex;		

*CAT* COPD assessment test, *CT* Computed tomography, *NBE* nodular bronchiectatic,* FC* fibrocavitary

a, interquartile range; b, Score for subjective symptoms of cough and sputum, included in CAT score.

**Table 2:** Reasons for sitafloxacin introduction and timing of initiation

Characteristic		No. of patients (*n* = 50)
Reason for STFX introduction; n (%)		
Persistent sputum culture positivity		20*(40)
Worsening chest CT findings		28 (56)
Lack of improvement on chest CT		11 (22)
Severe disease (initial concomitant use)		2 (4)
EB discontinuation		6 (12)
RFP discontinuation		3 (6)
Timing of STFX introduction; n (%)		
Initial concomitant use		7 (14)
Within 6 months of GBT initiation		2 (4)
After 6 months of GBT initiation		41 (82)
GBT initiation to STFX introduction interval; months, median (range)		14.7 (6.1–28.1)
STFX Treatment Duration		
Ongoing as of January 31, 2024; n (%)		22 (44)
Median duration in discontinued cases, months (range)		12.2 (6.8–19.1)
Reasons for STFX discontinuation		
Treatment completion (all medications)		10 (20)
Treatment failure		9 (18)
Adverse events		6 (12)
Patient request		1 (2)
Death		2 (4)

*STFX* sitafloxacin, *EB* ethambutol, *RFP* rifampicin, *GBT* guideline-based treatment

*Of the 20 patients, 16 also met the criterion of worsening chest CT findings, 3 overlapped with lack of improvement on chest CT, and 1 had concurrent difficulty with ethambutol use.

**Table 3:** Baseline treatment at sitafloxacin initiation

Characteristic		No. of patients (*n* = 50)
Number of GBT drugs in regimen (at baseline)		
2/3/4		20/27/3
Baseline drugs (generic name)		
Clarithromycin		34
Azithromycin		16
Ethambutol		44
Rifampin		35
Streptomycin		2
Amikacin		2

GBT, guideline-based treatment

### Assessment of treatment efficacy by group

Treatment outcomes for each group are summarized in Table 4. In Group 1, radiologic improvement was observed in 18.4% (9/49) of patients, subjective symptom improvement in 19.1% (9/47), and sputum culture conversion in 20% (4/20). The corresponding proportions in Group 2 were 12.5% (5/40), 20% (8/40), and 12.5% (2/16), respectively; in Group 3, 13.2% (5/38), 18.4% (7/38), and 13.3% (2/15); and in Group 4, 5.3% (1/19), 15.8% (3/19), and 12.5% (2/16), respectively.

**Table 4:** Evaluation of treatment efficacy across clinical subgroups

Characteristic		Group 1	Group 2	Group 3	Group 4
No. of patients		49	40	38	19
Age, years; median (range)^a^		68 (61–75)	67.5 (57.8–74.3)	67.5 (58.8–74.8)	68 (59.5–74.5)
Sex, female; n (%)		40 (81.6)	35 (87.5)	33 (86.8)	14 (73.7)
BMI (kg/m^2^); median (range)^a^		18.3 (16.4–19.6)	18.3 (16.6–19.6)	18.3 (16.5–19.6)	18.7 (16.6–19.7)
Radiographic pattern; n (%)					
Non-cavitary NBE type		28 (57.1)	25 (62.5)	23 (60.5)	8 (42.1)
FC type		6 (12.2)	4 (10)	4 (10.5)	4 (21.1)
Cavitary NBE type		15 (30.6)	11 (27.5)	11 (28.9)	7 (36.8)
Sputum Culture Analysis; n (%)					
Positive culture prior to STFX initiation		24 (49%)	19 (47.5%)	18 (47.4%)	19 (100%)
Culture conversion at 6–12 months post-STFX		4/20 (20%)	2/16 (12.5%)	2/15 (13.3%)	2/16 (12.5%)
Chest CT Assessment; median (range)^a^					
Baseline chest CT score before STFX initiation		9 (7–12)	9 (7.8–12)	9 (8–12)	9 (7.5–12)
Chest CT score at 5–7 months post-STFX		9 (6–12)	9 (7.5–12)	9 (8–12)	9 (8.5–12.5)
Patients with improved chest CT scores; n (%)		9/49 (18.4%)	5/40 (12.5%)	5/38 (13.2%)	1/19 (5.3%)
CAT score; median (range)^a^					
Baseline CAT score before STFX initiation		11 (6–18)	11 (6–18)	11 (6.3–18)	11 (6–19.5)
CAT score at 5–7 months post-STFX		12 (5.5–17.5)	13.5 (6.8–17.3)	14.5 (7–17.8)	16 (8.5–17.5)
Patients with improved CAT scores; n (%)		9/47 (19.1%)	8/40 (20%)	7/38 (18.4%)	3/19 (15.8%)

*BMI* Body Mass Index, *NBE* nodular bronchiectatic, *FC* fibrocavitary, *STFX* Sitafloxacin, *CT* Computed tomography

*CAT* COPD assessment test

a, interquartile range

Table 5 presents the characteristics of patients in Group 1 with improved chest CT findings. A significantly higher proportion of these patients had received STFX within six months of starting GBT (44.4% vs. 12.5%; *p* = 0.046). On univariable analysis, early administration of STFX (within six months) was associated with radiologic improvement (odds ratio [OR], 5.6; 95% confidence interval [CI], 1.114–28.143; *p* = 0.036; Table 6). However, among the nine patients who received early STFX, seven had it introduced as part of their initial treatment regimen. Therefore, the observed association may partly reflect the effect of initial therapy rather than salvage treatment, hence the potential for confounding must be considered.

**Table 5:** Characteristics of group 1 patients with improved chest CT findings

Characteristic		Improvement on chest CT	No improvement	*p* value
No. of patients		9	40	
Age, years; median (range)^a^		76 (67–77)	67.5 (57.8–73.3)	0.085
Sex, female; n (%)		7 (77.8)	33 (82.5)	0.663
BMI (kg/m^2^); median (range)^a^		18.3 (16.2–18.7)	18.3 (16.6–19.6)	0.992
Absence of cavitary lesions; n (%)		5 (55.6)	23 (57.5)	1.000
Early STFX Introduction*; n (%)		4 (44.4)	5 (12.5)	**0.046**
EB co-administration; n (%)		8 (88.9)	35 (87.5)	1.000
CLA-susceptible strain; n (%)		7/7 (100)	30/35 (85.7)	0.569
*M. avium*, n (%)		8 (88.9)	32 (80)	1.000
STFX initiation with negative sputum culture**; n (%)		6 (66.7)	19 (47.5)	0.463
STFX-induced culture conversion at 6–12 months***; n (%)		1/3 (33.3)	3/17 (17.6)	

*BMI* Body Mass Index, *STFX* sitafloxacin, *EB* ethambutol, *CLA* clarithromycin, *M. avium* *Mycobacterium avium*

*Introduction of sitafloxacin within six months of guideline-based treatment initiation

**Patients with negative sputum culture at the time of STFX initiation

***Patients who achieved sputum culture conversion 6–12 months after STFX initiation

**Table 6:** Univariate logistic regression analyses of factors associated with improved chest CT findings in group 1

	Univariate logistic regression		
Variable	OR	95%CI	*p*-value
Age, years	1.062	0.982–1.148	0.13
Sex, female	0.742	0.126–4.361	0.742
BMI (kg/m^2^)	1.002	0.737–1.360	0.992
Early STFX initiation*	5.6	1.114–28.143	**0.036**
EB co-administration	1.143	0.117–11.177	0.909
Absence of cavitary lesions	0.924	0.215–3.965	0.915
Negative sputum culture	2.211	0.484–10.092	0.306
*M. avium*	0.50	0.054–4.597	0.54

*BMI* Body Mass Index, *STFX* sitafloxacin, *EB* ethambutol, *M. avium*,* Mycobacterium avium*

*Introduction of sitafloxacin within six months of guideline-based treatment initiation

Table 7 presents the characteristics of Group 1 patients who achieved sputum culture conversion; however, no variables were significantly associated with this outcome.

**Table 7:** Characteristics of group 1 patients with sputum culture conversion

Characteristic		Culture conversion	No conversion	*p*-value
No. of patients		4	16	
Age, years; median (range)^a^		74.5 (68.3–80.3)	66.5 (72.8–57.8)	0.219
Sex, female; n (%)		2 (50)	12 (75)	0.549
BMI (kg/m^2^); median (range)^a^		18.6 (17.5–18.9)	18.8 (16.6–20.4)	0.748
Absence of cavitary lesions; n (%)		3 (75)	4 (25)	0.101
Early STFX Introduction; n (%)		2 (50)	2 (12.5)	0.162
EB co-administration; n (%)		3 (75)	14 (87.5)	0.509
CLA-susceptible strain; n (%)		4 (100)	13 (81.3)	1.000
*M. avium*, n (%)		3 (75)	11 (68.8)	1.000

*BMI* Body Mass Index, *STFX* sitafloxacin, *EB* ethambutol, *CLA* clarithromycin; *M. avium*,* Mycobacterium avium.*

Table 8 summarizes the clinical, radiologic, and microbiological data of patients in Group 4 with MAC-PD following STFX treatment initiation. Among the two patients (#2,#12) who achieved sputum culture conversion, both had CLA-susceptible strains at baseline, received EB, and exhibited non-cavitary disease.

**Table 8:** Changes in sputum culture results and drug susceptibility among patients in group 4

Case number	Sputum culture conversion	Sex	Age, years	Sputum examination results by Timeline								Baseline MIC [CLA] (µg/mL)	Baseline MIC [LVFX] (µg/mL)	6–12 months post-MIC [CLA] (µg/mL)	6–12 months post-MIC [LVFX] (µg/mL)	Drugs at STFX initiation	Radiologic pattern
				6 months pre-STFX (Baseline)				6–12 months post-STFX initiation									
				Count	Culture	PCR	Smear	Count	Culture	PCR	Smear						
1	−	F	86	4	2	4	4	3	1	3	3	> 16	0.5	> 32	8	C, E, R	FC type
2	་	F	70	3	1	0	0	3	0	0	0	0.25	2			C, E, R	Non-cavitary NBE type
3	−	F	71	2	1	1	0	5	1	0	0	16	0.5	4	0.5	C, E	Non-cavitary NBE type
4	−	F	52	3	2	2	3	2	1	1	0	> 32	0.5	> 32	8	C, E, A	Cavitary NBE type
5		M	74	2	1	1	1	1	0	0	0	2	4			Z, E, R	Cavitary NBE type
6	−	F	56	4	1	2	0	2	1	2	0	0.125	1	0.25	2	C, E, R	Cavitary NBE type
7	−	F	82	2	1	1	1	5	4	4	3	1	1	> 32	16	C, E	FC type
8	−	F	68	1	1	0	0	1	1	1	1	0.125	0.5	0.125	1	C, R	Non-cavitary NBE type
9	−	M	52	2	1	1	0	2	2	2	2	0.25	4	2	4	C, E	FC type
10	−	F	57	3	1	0	0	2	1	1	1	16	1	8	1	C, E, R	Cavitary NBE type
11	−	M	58	2	1	2	2	2	2	2	1	> 32	4	> 32	4	C, E, R	Cavitary NBE type
12	་	F	63	2	2	2	0	2	0	0	0	0.5	4			C, E, R	Non-cavitary NBE type
13	−	F	66	2	1	1	0	2	1	0	0	1	4	0.5	2	C, E, R	Non-cavitary NBE type
14	−	M	62	1	1	1	1	2	2	2	2	1	2	1	4	C, E, R, S	Cavitary NBE type
15	−	F	79	3	1	1	1	4	1	1	0	0.06	1	0.5	4	C, E, R	FC type
16	−	F	61	3	3	3	3	2	2	2	2	> 32	1	> 32	2	C, E, R, A	Cavitary NBE type
17	−	F	75	3	2	3	2	3	3	3	3	0.5	0.5	0.5	0.5	C, E, R	Non-cavitary NBE type
18		F	70	2	1	1	1	1	0	0	0	1	2			C, E, R	Non-cavitary NBE type
19		M	80	3	1	1	0	1	0	0	0	2*	0.25 (STFX)*			Z, E, R	Non-cavitary NBE type

*M* male, *F* female, *MIC* minimum inhibitory concentration, *CLA* clarithromycin, *LVFX* Levofloxacin, *STFX* sitafloxacin, *C* clarithromycin, *E* ethambutol, *R* rifampicin, *S* streptomycin, *A* amikacin, *Z* azithromycin, *NBE* nodular bronchiectatic, *FC* fibrocavitary

*All MICs were measured using BrothMIC NTM, except this value, which was obtained using BrothMIC SGM.

## Discussion

This study evaluated the therapeutic impact of STFX-containing regimens administered for ≥ 6 months in patients with MAC-PD. Across all clinical subgroups, rates of sputum culture conversion, radiologic improvement, and symptom relief consistently remained below 20%, indicating limited overall efficacy.

In contrast, two previous studies documented more favorable outcomes with STFX. Fujita et al. reported culture conversion, radiologic improvement, and symptom relief rates of 44.4%, 55.8%, and 70%, respectively, in 18 patients treated for at least 4 weeks [16]. Similarly, Asakura et al. observed corresponding rates of 23%, 19%, and 26% in patients with refractory MAC-PD [17]. However, both studies employed shorter treatment durations (≥ 4 weeks) compared to this study (≥ 6 months), which may have led to an overestimation of the clinical effectiveness of STFX.

Key differences in patient characteristics among the studies by Fujita et al., Asakura et al., and this study were as follows: CLA resistance rates (not reported/48%/14%), proportion of cavitary lesions (55.6%/42%/44%), median BMI (18.7/18.1/18.3), EB co-administration rates (44.4%/71%/88%), median time from GBT initiation to STFX addition (not reported/60 months/14.7 months), and persistent sputum culture positivity at STFX initiation (100%/100%/40%). Based on these comparisons, our cohort appeared to have a more favorable baseline profile for treatment success: the proportion of CLA-susceptible isolates was higher, EB co-administration was more frequent, and STFX was introduced earlier relative to GBT initiation. Additionally, the proportion of patients with cavitary lesions and the median BMI were generally comparable across studies. It should also be noted that our study applied a less stringent definition of sputum culture conversion—two consecutive negative cultures at least four weeks apart—whereas previous studies used the conventional criterion of three consecutive negative cultures [18]. Despite favorable clinical characteristics and a more lenient methodologically different definition of culture conversion, treatment success rates in our cohort were lower than those previously reported. Although small sample sizes may have contributed to variability in outcomes, the underlying reasons for this discrepancy remain unclear.

Group 4, comprising patients with refractory MAC-PD and persistent sputum culture positivity, demonstrated a sputum culture conversion rate of only 12.5% (2/16) using the definition of two consecutive negative cultures. In contrast, ALIS has shown greater efficacy in refractory MAC PD. The CONVERT trial reported a 6-month culture conversion rate of 29% [6], and real-world data from Japan demonstrated rates of 58.3% [19] and 56.8% [20]. In addition, culture conversion was 8.9% (10/112) in the CONVERT GBT-alone arm under a three-negative criterion [6], compared with 12.5% (2/16) in our refractory subgroup treated with GBT plus STFX using the two-negative criterion. Although cross-trial comparisons should be interpreted with caution, these findings suggest that adding STFX to GBT confers, at best, limited incremental benefit. Compared to these outcomes, STFX appears less effective and is unlikely to be suitable as a first-line agent in the treatment of refractory MAC-PD. Its use may be limited to patients in whom aminoglycosides are contraindicated or poorly tolerated.

In this study, early initiation of STFX, defined as within 6 months of GBT initiation, was the only factor independently associated with radiologic improvement in Group 1. However, in most cases of these cases STFX was introduced as part of the initial treatment regimen. Thus, the observed benefit may be partially attributable to the effects of initial therapy rather than STFX alone. In Group 2, which excluded patients who received early STFX initiation and included those with more refractory disease, no significant predictors of radiologic improvement were identified (data not shown). These findings suggest that while early introduction of STFX may offer some clinical benefit, this should be interpreted with caution, due to potential confounding factors. Notably, previous studies have also reported an association between improved outcomes and the early use of FQ-containing regimens. For example, a gatifloxacin-containing regimen achieved a culture conversion rate of 84.6% [10], and a randomized trial comparing RFP/EB/CAM with RFP/EB/ciprofloxacin demonstrated comparable cure rates [11]. However, other studies have reported that early addition FQs to GBT did not significantly improve culture conversion rates [21], underscoring the uncertainty regarding their clinical benefit.

Recent studies have cautioned that substituting EB with FQs may reduce treatment efficacy [22, 23] and increase the risk of CLA resistance when EB is omitted from macrolide–FQ regimens [24]. These findings highlight the importance of maintaining EB co-administration when using FQ-based therapies, including STFX. In our study, previously recognized negative prognostic indicators—such as absence of EB co-administration, cavitary disease [25], CLA resistance [24], and *M. intracellulare* infection [26]—were not significantly associated with treatment response. However, among Group 4 patients with persistent sputum culture positivity, the two individuals who achieved culture conversion shared three favorable features: CLA-susceptible isolates, EB co-administration, and non-cavitary disease. These observations suggest that STFX may be most effective in a distinct subgroup of patients who exhibit this triad of clinical factors.

This study has several limitations. First, this was a retrospective single-center study with a limited sample size, which may affect the generalizability of the findings. Second, the standard definition for sputum culture conversion could not be fully applied because sputum sampling was insufficient in some cases. Third, minimum inhibitory concentration (MIC) data for STFX were unavailable, as susceptibility testing at our institution during the study was conducted using the BrothMIC NTM^®^ Far East system, which does not include STFX. Fourth, in Group 1, only nine cases showed radiologic response at six months. This small number of events per variable limited the feasibility of multivariable modeling, therefore, univariable associations, as reported here, should be regarded as primarily exploratory and potentially confounded. Fifth, the safety of STFX could not be comprehensively assessed because only patients who continued treatment for ≥ 6 months were included, and adverse events not resulting in discontinuation were not recorded systematically. Finally, comparative analysis between EB-intolerant and RFP-intolerant patients was not feasible due to the limited number of cases and the clinical similarity between these groups (Supplementary Table 2).

## Conclusions

STFX-containing regimens demonstrated limited efficacy in patients with MAC-PD, with response rates below 20%. Early introduction of STFX may offer some clinical benefit, however, its overall impact remains uncertain. STFX should thus be considered only when standard treatment options are not feasible.

## Supplementary Information

Supplementary Material 1.

Supplementary Material 2.

## Data Availability

All data generated or analyzed during this study are included in this article. Further inquiries can be directed to the corresponding author.

## Abbreviations

ALIS: Amikacin liposome inhalation suspension
ATS: American thoracic society
AZM: Azithromycin
BMI: Body mass index
CAT: COPD assessment test
CLA: Clarithromycin
CT: Computed tomography
EB: Ethambutol
ESCMID: European society of clinical microbiology and infectious diseases
ERS: European respiratory society
FC: Fibro-cavitary
FQ: Fluoroquinolone
GBT: Guideline-based treatment
IDSA: Infectious diseases society of America
MAC: Mycobacterium avium complex
MAC-PD: Mycobacterium avium complex pulmonary disease
MCID: Minimal clinically important difference
MIC: Minimum inhibitory concentration
NBE: Nodular bronchiectasis
NICE: Nodule, infiltration, cavity, ectasis scoring system
NTM: Nontuberculous mycobacteria
RFP: Rifampicin
SM: Streptomycin
SPSS: Statistical package for the social sciences
STFX: Sitafloxacin
